# Ascaris lumbricoides a rare cause gastric perforation: a case report and brief literature review

**DOI:** 10.3389/fmed.2024.1525301

**Published:** 2025-01-08

**Authors:** Tian-Hao Xie, Yan Fu, Xiang-Xiang Ren, Xin-Li Sun, Qiang Wang, Qian Sun

**Affiliations:** ^1^Department of General Surgery, Affiliated Hospital of Hebei University, Baoding, Hebei, China; ^2^Basic Research Key Laboratory of General Surgery for Digital Medicine, Baoding, Hebei, China; ^3^Department of Ophthalmology, Baoding No.1 Central Hospital, Baoding, Hebei, China

**Keywords:** ascariasis, gastric perforation, complication, case report, review

## Abstract

*Ascaris lumbricoides* (AL), a prevalent nematode causing ascariasis, infects millions worldwide, with a higher risk in preschool and school-aged children. Though infections are usually mild, rare and life-threatening complications like gastrointestinal perforation exist. This article documents a case involving a 61-year-old deaf-mute man who presented with a month-long history of epigastric pain accompanied by nausea, anorexia, and constipation. The pain exacerbated, eventually extended to encompass the entire abdomen 4 h prior to being diagnosed with hollow viscus perforation. During the surgical procedure, three live ascarids were discovered within the abdominal cavity, and the jejunum was found to be filled with a large number of ascarids, accompanied by a perforation in the gastric antrum. Subsequently, the ascarids were extracted, and the perforated area was repaired. Postoperatively, the patient underwent anti-infection therapy, acid suppression, gastric mucosa protection, and nutritional support. On postoperative day (POD) 1, a single dose of 400 mg albendazole was administered. Fecal samples on POD 3, 5, and 6 tested positive for AL eggs. The patient recovered smoothly with no evidence of peptic ulcer disease on one-month follow-up endoscopy. Additionally, fecal tests conducted over three consecutive days did not detect any AL eggs. This case highlights the crucial importance of recognizing ascariasis-associated complications and underscores the paramount role of timely surgical intervention in such cases. Meanwhile, this article combines cases of gastrointestinal perforation caused by AL documented in the literature since 1903, elaborates on the epidemiological characteristics, pathogenesis, diagnosis, treatment, and prevention of ascariasis, and analyzes the reasons for the occurrence of such complications.

## 1 Introduction

*Ascaris lumbricoides* (AL), commonly known as the human intestinal roundworm or simply the roundworm, is a nematode that is a major cause of ascariasis in humans. AL infects approximately 820 million people and is prevalent in at least 103 of the 218 countries in the world (1). This parasitic infection typically occurs in the small intestine, where the worms can grow up to 35 cm (14 inches) long. AL infections, which pose a higher risk of infection in preschool-aged and school-aged children, are usually acquired through the ingestion of food or water contaminated with fecal matter containing the parasite’s eggs (2).

AL infections are usually mild, although severe complications, although rare, still exist (3). The symptoms of ascariasis can vary depending on the number of ascarids present in the intestine and the individual’s immune response (2). Common symptoms include abdominal pain, intestinal obstruction, nausea, vomiting, diarrhea, weight loss, and malnutrition. In some cases, the ascarids may migrate out of the intestine and cause obstruction in other parts of the body, such as the bile duct or appendix, leading to conditions like cholangitis, obstructive jaundice, pancreatitis, or appendicitis (4). The digestive tract perforation as a fatal complication of AL is extremely rare, with only 14 cases reported in previous literature (5–15), 3 of which were gastric perforations (5, 11, 12) (Table 1). Herein, we report a case of gastric perforation in a patient with ascariasis confirmed by surgery. In addition, we analyzed this rare complication in combination with previous literature.

**Table 1:** Overview of documented cases.

Author (year)	Sex	Age	Special personal history	Chief complaints	Concomitant symptom	Perforation site	Outcome
Castor (1903) (5)	Male	43	Take drugs	Abdominal pain	Inappetite, irregularity of bowels, anemia	Gastric	Death
	Male	26	Take drugs	Abdominal pain	Loss weight, fever	Ileum	Death
Ihekwaba FN (1979) (6)	Male	6	Vomited worms	Abdominal distension	Nausea, inappetite	Ileum	Recovered
	Male	8	Vomited worms	Abdominal pain	Vomiting, fever, inappetite	Ileum	Recovered
	Male	8	Vague abdominal pain	Abdominal pain	Vomiting, fever, inappetite	Ileum	Recovered
Cotton (1987) (7)	Female	3	Vomited worms	Intestinal obstruction	NM	Intestinal	NM
Paraskevaides (1988) (8)	Male	58	NM	Abdominal pain	Nausea, fever	Intestinal	NM
Refeidi (2007) (9)	Male	35	NM	Epigastric pain	Nausea, anorexia, constipation, vomiting, fever	Duodenal	Recovered
Sarmast (2011) (10)	Female	35	Peptic ulcer	Epigastric pain	Nausea	Duodenal	Recovered
Gupta (2012) (11)	Male	48	Passed worms in stools	Epigastric pain	Vomiting, fever	Gastric	Recovered
Ntirushwa (2016) (12)	Female	19	Epigastric pain	Abdominal distension and pain, fever	Cesarean delivery 4 days ago	Gastric	Death
Molina (2018) (13)	Female	55	Unremarkable	Abdominal pain	Malnutrition	Colon	Recovered
Darlington (2018) (14)	Male	4	Vomited worms, passed worms in stools, pica	Abdominal distension and pain	Constipation, inappetite	Ileum	Recovered
Molla (2023) (15)	Female	2	Vomited worms	Abdominal distension	Vomiting, inappetite	Ileum	Recovered
Personal case	Male	61	Unremarkable	Abdominal pain	Nausea, anorexia, constipation	Gastric	Recovered

NM, Not mentioned.

## 2 Case report

A 61-year-old deaf-mute male patient was admitted to our emergency department with a one-month history of epigastric pain associated with nausea, anorexia, and constipation. The pain intensified and spread to involve the entire abdomen 4 h ago. He was unmarried and lived alone in a remote village. He had neither traveled to an infected area nor had a history of digestive ulcers. Furthermore, the patient had no known medical, surgical, family, psychosocial, or medication history. The patient exhibited signs of distress but was able to give correct feedback during the physical examination. His body temperature was 37.1°C, blood pressure was 109/63 mmHg, pulse rate was 96 beats per minute, and respiratory rate was 27 breaths per minute. Abdominal examination revealed a flattened abdomen with board-like rigidity upon palpation. The result of abdominal auscultation was that no bowel sounds were audible.

**Figure 1:**
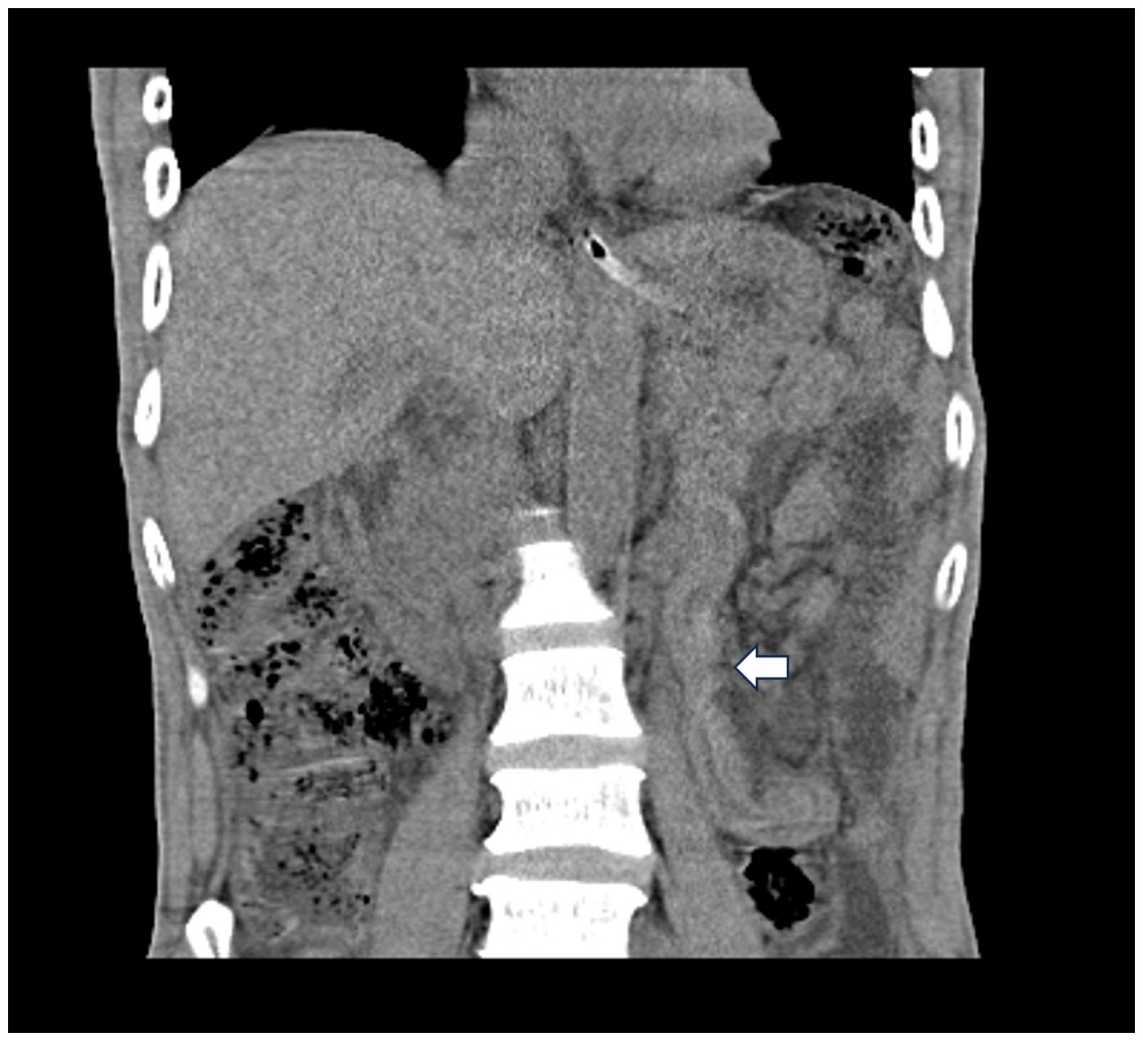
Plain computed tomography of the abdomen revealed a stringy shadow in the jejunum (white arrow).

The white blood cell count was 15.07 × 10^9^/L, hemoglobin was 118 g/L, neutrophil count was 14.06 × 10^9^/L, neutrophil percentage was 93.3%, serum sodium was 131 mmol/L, blood urea nitrogen was 8.2 mmol/L, fibrinogen was 5.85 g/L, serum procalcitonin was 4.74 ng/mL and blood glucose was 7.75 mmol/L. The remainder of the blood tests revealed no significant abnormalities. Plain computed tomography (CT) of the chest revealed pneumonia in the left lung. Plain CT of the abdomen revealed a small amount of fluid and free air in the abdominal cavity, as well as stringy shadow in the jejunum (Figure 1).

**Figure 2:**
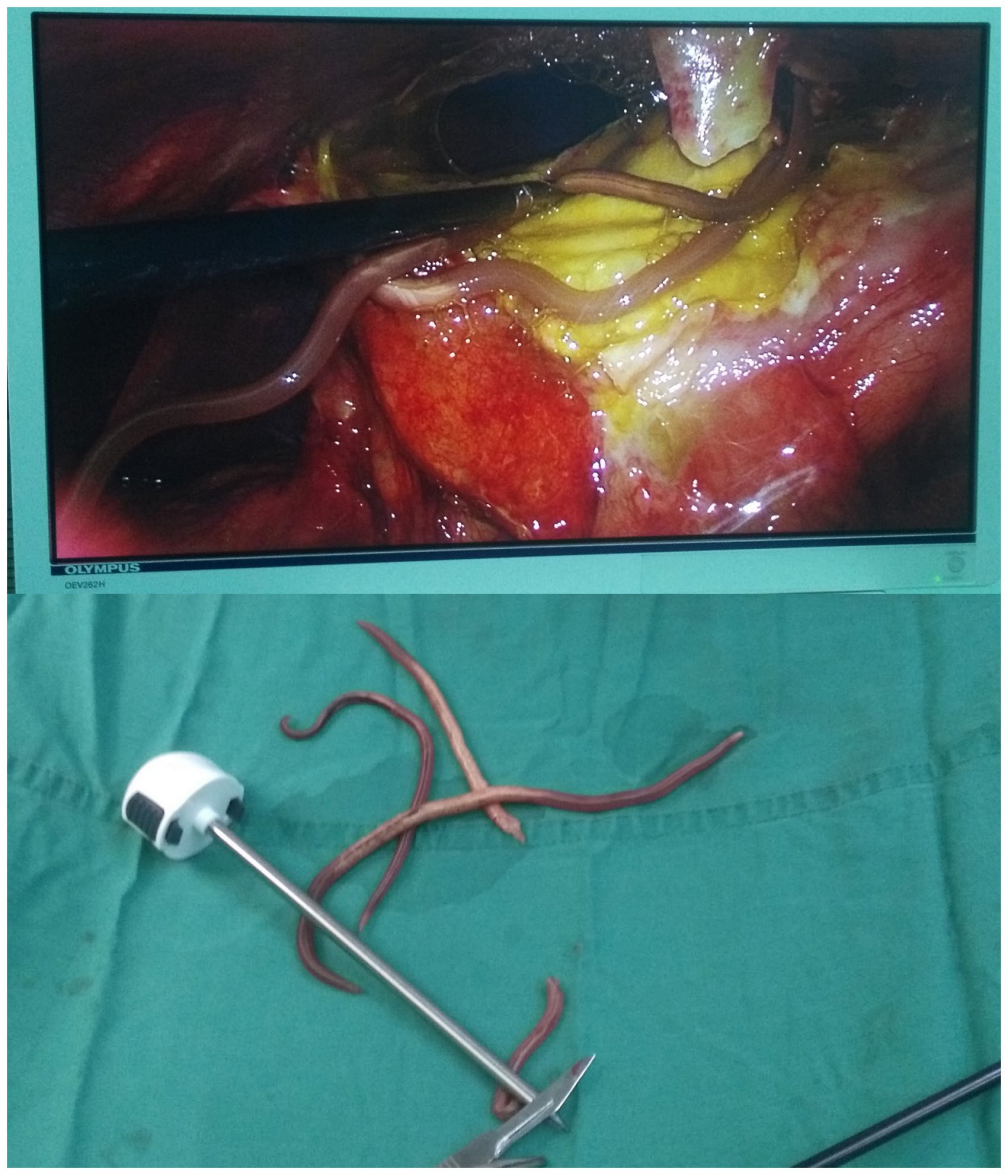
Three viable ascarids were identified within the abdominal cavity under laparoscopic exploration.

Hollow viscus perforation was diagnosed based on the reported clinical symptoms and auxiliary examination, and active resuscitation was initiated in preparation for surgery. Laparoscopic exploration was carried out under general anesthesia. In addition to 700 mL of pus in the abdominal cavity and pus mosses on the surface of the stomach, 3 viable ascarids were finding around pus mosses (Figure 2). In order to accurately examine the stomach and the small intestine, the surgical procedure was changed to exploratory laparotomy. The 3 live ascarids were removed and the pus was drained. There was a perforation (about 7 mm in diameter) in the gastric antrum after removing the pus mosses. Except for the gastric tube, there was no strip-like object could be palpated in the stomach cavity. There was no scarring or induration around the perforation to suggest a long-standing peptic ulcer disease.

**Figure 3:**
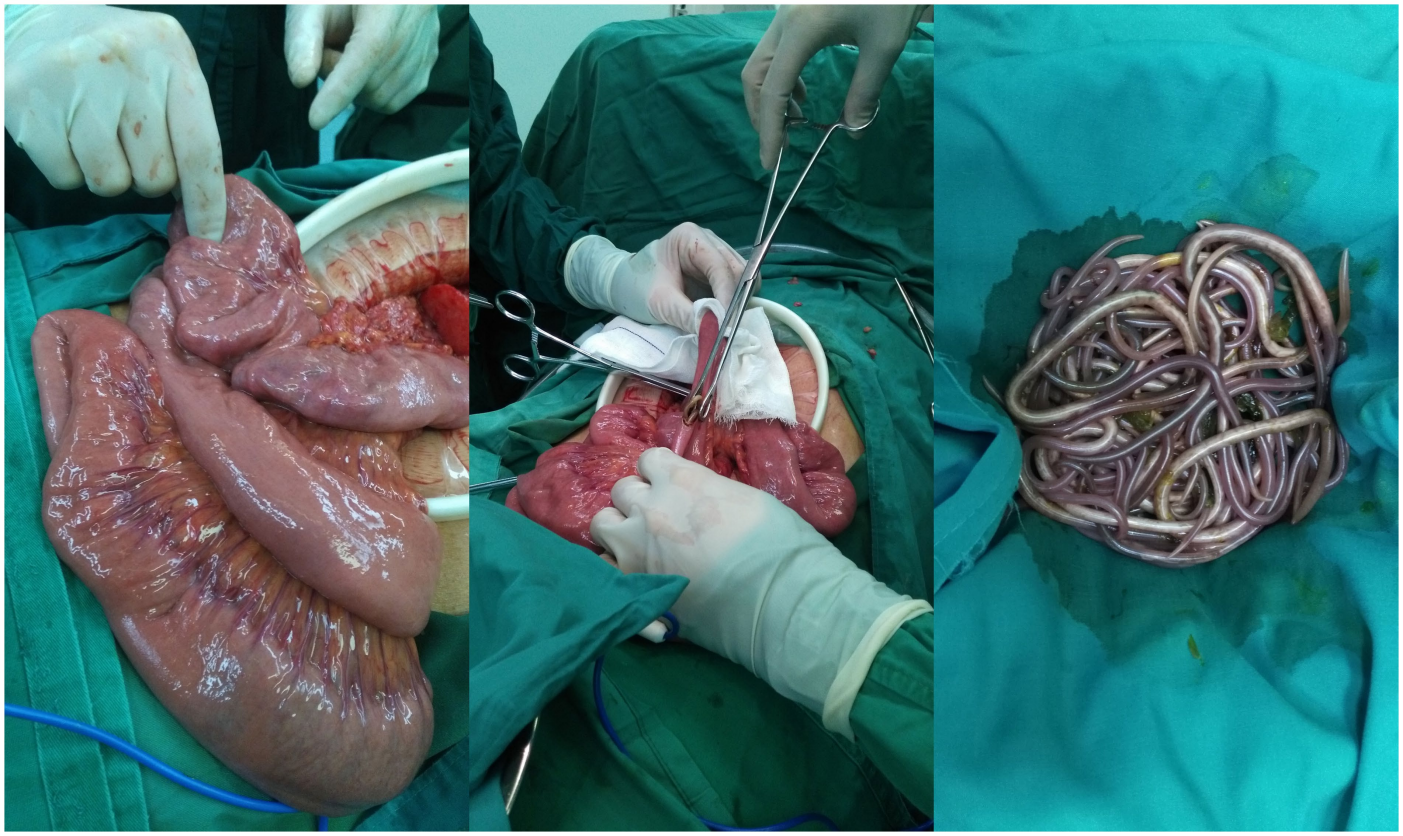
The ascarids in the jejunum were removed.

Subsequently, the perforation was closed using a prolene non-absorbable stitch. Continuing to explore the small intestine, a bundle of ascarids could be palpated within the jejunum. The ascarids was removed after the jejunum was cut open, and the intestinal wall was closed with a closure device (Figure 3). The peritoneal cavity was washed thoroughly with a lot of warm saline. The abdomen was closed routinely after 3 suction drains placed in the abdominal cavity and pelvic cavity.

Postoperatively, the patient received anti-infection therapy, acid suppression, protection of the gastric mucosa, nutritional support and other related treatments. Adjuvant medical therapy with a single dose of 400 mg albendazole was administered on postoperative day (POD) 1. Fecal samples collected from the patient on POD 3, as well as on POD 5 and POD 6, all tested positive for AL eggs. On POD 10, the patient recovered smoothly and was discharged. Afterwards, the patient continued to take oral Omeprazole. An upper gastrointestinal endoscopy performed 1 month later did not show any evidence of peptic ulcer disease. Additionally, fecal tests conducted over three consecutive days did not detect any AL eggs. The timeline is described in Table 2.

**Table 2:** The timeline of the diagnosis and treatment process.

Timeline	Diagnosis and treatment process
Dec 29th, 2023	Epigastric pain associated with nausea, anorexia, and constipation
Jan 31th, 2024	The pain intensified, and the patient was hospitalized in the department of General Surgery
Feb 1th, 2024	Surgery was performed, and gastric perforation caused by AL was confirmed
Feb 2th, 2024	Albendazole was administered
Feb 4th, 2024	Fecal samples tested positive for AL eggs
Feb 6th, 2024	Fecal samples tested positive for AL eggs
Feb 7th, 2024	Fecal samples tested positive for AL eggs
Feb 13th, 2024	Discharged from the hospital
Mar 16th, 2024	The results of fecal tests and upper gastrointestinal endoscopy were negative

AL, Ascaris lumbricoides.

## 3 Discussion

### 3.1 Epidemiology

AL, originally described by Carl Linnaeus in 1758, is the causative agent of the human disease ascariasis. The World Health Organization (WHO) has confirmed that AL is one of the most prevalent human *soil-transmitted helminths* (STHs), including *Trichinella*, *Ancylostoma duodenalis*, and *Necator americanus* (16).

Ascariasis is a widely common parasitic disease worldwide, especially in countries or regions where the economy is not developed, the climate is warm and humid, and poor health conditions prevail. The majority of infections occur in tropical and subtropical regions of the world, with Sub-Saharan Africa, the Americas, China, and East Asia recording the highest prevalences (17).

China was once one of the countries with the most serious prevalence of STHs in the world, which seriously affected the health of the Chinese people and hindered social and economic development. From 1988 to 1992 (18), 2001–2004 (19), 2014–2016 (20), and from 2019 to 2020 (21), China has carried out the national human parasite distribution survey four times. According to the survey results in 2020, the infection rate of AL was 0.19%. Tibet had the highest rate (1.64%) (22). Beijing, Hebei, Inner Mongolia, Heilongjiang, Shanghai, Zhejiang, Fujian, and Shaanxi have not reported any AL infections; the infection rates in the other 21 provinces (municipalities and autonomous regions) were all below 1.00%. Men and women infection rate was 0.19%; the infection rate of 7–14 years old group was the highest (0.26%). Over 60 age group the lowest prevalence, 0.17%. According to the results of the national survey on the status of major human parasitic diseases in 2015, 4,343 cases of AL infections were detected in 31 provinces (municipalities and autonomous regions), with a weighted infection rate of 1.36%, and the number of AL infections in China was estimated to be 8.82 million (20). It is second only to hookworm in STHs infections (22).

### 3.2 Morphology

Adult ascarids have long, smooth, cylindrical bodies ranging from several centimeters to over 30 cm, covered with a protective cuticle (23). They have a small mouth surrounded by lips and labial papillae for grasping food, as well as a simple digestive tract. They are hermaphroditic, with reproductive organs in the posterior region. The body tapers to a pointed tail for locomotion. Color varies by species and the contents of the host’s intestine (24). AL eggs are oval or elliptical, measuring 45–75 μm in length and 35–50 μm in width, encased in a tough, transparent chorion. They contain an embryo and polar granules, which are crucial for development. Freshly laid eggs are colorless or yellowish-white, and they turn brownish over time. The egg’s surface may have a fine granular or reticulate pattern, observable under a microscope (25).

### 3.3 Life cycle

AL does not require an intermediate host to complete its life cycle. Instead, it has a direct life cycle that involves humans as the definitive host. The life cycle of AL begins with the female ascarids producing eggs, which are then passed out in the feces of the infected human. These eggs are subsequently released into the environment, often contaminating soil or water. After 5–10 days of development in moist, shady, oxygen-rich soil at 21–30°C (while at 17°C it can take 45–55 days), the embryonic cells in the fertilized eggs hatch and develop into first-stage larvae (L1) (26). The larvae then undergo ecdysis twice, developing into second-stage (L2) and then third-stage (L3) larvae, which are infective. Both the first and second ecdysis occur before the eggs hatch in the large intestine, and the retention of two ecdysis sheaths is thought to be a trait conducive to parasite development (27).

Human beings are infected by these worms when they consume food or water that is contaminated by mature Ascaris worm eggs containing L3 larvae. Once inside the human body, the larvae migrate to the liver via the portal blood vessels, where the L2 cuticle is shed and some larval growth occurs. Subsequently, L3 larvae leave the liver and enter the lungs through the bloodstream, first reaching the heart and then the pulmonary vessels (26, 28). In the lungs, larvae cross the alveolar space and then migrate along the airway tree to the pharynx, where they are coughed up and swallowed. As they return to the small intestine, L3 larvae undergo ecdysis to become the fourth larval stage (L4 larvae), which then undergoes a final ecdysis (L5) and develops into adult and sexually mature male and female ascarids (28, 29). These ascarids produce eggs that are passed out in the feces, completing the life cycle. Therefore, unlike some other parasites that require intermediate hosts to complete their life cycles, AL does not need an intermediate host and can complete its entire life cycle within a single human host.

### 3.4 Pathogenic mechanism

Both larvae and adults of parasites are capable of inducing diseases, manifesting as mechanical damage, hypersensitivity, malnutrition, intestinal dysfunction, among others, and they also impact the microbiota indirectly by modifying the host’s physiology and immune system (30).

Migration of small numbers of larvae into the lungs generally does not cause obvious symptoms. But a large number of larvae may trigger mechanical injury and toxic effects during migration, which may lead to thin bronchial epithelial cell falls off, lung bleeding point, causing bronchopneumonia, bronchial asthma, or eosinophilia. The molting of the larvae and its molting fluid may also act as an irritant, causing an immediate allergic reaction and further aggravating asthma symptoms (17). The incubation period is generally 1–9 days, during which transient respiratory symptoms may appear, with a duration of no more than 4 weeks. The main clinical manifestations are cough, chest tightness, throat itching, dry cough, asthma, and urticaria, occasionally accompanied by fever, bloody sputum, or allergic dermatitis (31). X-ray examination showed that the enlargement of hilar shadows and increased lung marking, accompanied by punctate, flocculent or flaky shadows. These imaging findings resolved after 1–2 weeks. During this period, eosinophils or AL larvae are often found on sputum smears. In severe cases, the larvae invade the brain, liver, spleen, kidney, eye and thyroid gland, and even enter the fetus through the placenta to cause ectopic parasisis (17, 19, 32).

Adult ascarids parasitize the jejunum, feed on semi-digested food in the small intestine, rob the host of nutrients, and damage the intestinal mucosa, which leading to dyspepsia and malabsorption of nutrients, and even malnutrition in severe cases (23). Children serious infection can cause developmental disability. Symptoms include loss of appetite, nausea, vomiting, abdominal pain, bloating (33). After entering the body, the ascarids may release toxins or mechanically irritate the bowel wall, causing abnormal gastrointestinal motility, which can lead to nausea and vomiting (34). When patients experience nausea, they may feel a foreign body sensation or discomfort in the throat, and vomiting may be sudden and intense. The abdominal pain is characterized by intermittency, often located around the umbilicus, recurrent episodes, and spontaneous relief. Children are often accompanied by neuropsychiatric symptoms such as convulsions, night terrors, bruxism, and occasionally pica (35, 36). Adult ascarids can also cause hypersensitivity reactions such as urticaria, pruritus, conjunctivitis and toxic encephalopathy. This may be due to the induction of IgE mediated by ascarids allergen (37).

“Drilling holes” is an important biological behavior of AL (38). In general, ascarids do not actively “drill holes” in the traditional sense, as this behavior is not a common or defining characteristic of the entire group (39). However, this behavior is not specific to “drilling holes” but rather a survival strategy for finding a suitable living environment or food source within the host (40). This migration may give the appearance of “drilling holes,” but it is actually a part of their parasitic lifestyle. Under the stimulation of fever, gastrointestinal disease, eating spicy food or inappropriate dosage of anthelmintic drugs and other factors, ascarids migrate to the bile ducts, pancreatic ducts, appendix, and even the liver through an opening in the wall of the intestine (4, 41).

It has also been reported in the literature that adult ascarids migrate to the trachea or bronchi, causing airway obstruction and even asphyxia (31). Biliary ascariasis is the most common clinical complication, which can cause hemobilia, liver abscess, cholelithiasis, gallbladder rupture and biliary peritonitis (42). Ascarids accumulates in the intestine and further progresses to intestinal obstruction, volvulus, intussusception, and intestinal necrosis (43). Severe cases may occur intestinal perforation and acute peritonitis, case fatality rate is high (13, 44).

### 3.5 Diagnosis

Etiological examination is the basis for diagnosis. Kato-Katz smear is the WHO recommendation, a kind of most widely used international dung egg method, suitable for qualitative and quantitative analysis of eggs (17). Because the ascarids produces a large number of eggs, a female ascarids can lay up to 200,000 eggs in the small intestine each day. Using the smear method, the detection rate of the eggs can reach 95% with just three smears. However, owing to the polymorphic nature of AL eggs and the presence of debris that can cause interference under microscopic examination, non-parasitic elements such as pollen, plant cells, and mites are occasionally misinterpreted as AL eggs (45). In such scenarios, sophisticated techniques such as the saturated salt water floatation method or sedimentation methods (including the likes of (Mini-)FLOTAC, McMaster, and FECPAKG2) are invaluable in precisely separating debris from the eggs, ultimately enhancing the overall detection rate with remarkable accuracy (46).

When ectopic ascariasis arises due to larval migration, accompanied by concurrent infections, there may be an elevation in white blood cell and eosinophil counts in the blood, albeit these findings are not definitive or specific in isolation (47, 48).

In certain instances, ultrasound is capable of identifying ascarids, which may manifest as a prominent echogenic mass or a distinct elongated echogenic band. These echogenic signatures may exhibit features like acoustic shadowing or visible movement (48, 49). However, diagnostic accuracy can be compromised due to factors such as intestinal gas and fecal matter, which tend to degrade the quality of the ultrasound images.

The barium meal X-ray examination offers a clear visualization of the ascarids’ morphology and quantity, greatly assisting in assessing the severity of ascariasis (50). In instances where ascarids inhabit the stomach, the examination reveals variable round-strip shadows resembling the ascarids’ size (51). If several worms align in parallel, their shadows will exhibit a distinct “rice grain-like” pattern, while the cross-sectional projections of the worms’ bodies will appear as the “strip,” “four-lines,” “inner-tube,” “double-tube,” “bull’s-eye,” “target,” or “zig-zag” sign, and are described as a “worm mass” or “spaghetti-like” appearance, providing valuable insights into the presence and extent of the infestation (15, 52).

CT scanning provides a clear visualization of the ascarids’ morphology and precise location, significantly contributing to the diagnosis of ascariasis and the assessment of its potential complications (52). On CT images, the presence of ascarids is typically characterized by punctate or linear soft tissue density shadows within dilated bile ducts or the gastrointestinal tract, offering invaluable insights for clinical management (40, 53).

Endoscopic techniques, particularly the utilization of fiberoptic and electronic gastroduodenoscopy, have revolutionized the diagnosis and treatment of ascarids in the stomach or duodenum (4, 54, 55). These techniques enable physicians to visually detect the worms directly, using precision tools such as grasping forceps, snares, and baskets to extract them in a single, comprehensive procedure. Furthermore, endoscopic retrograde cholangiopancreatography (ERCP) accurately pinpoints the location of ascarids within the biliary tract, facilitating their removal with specialized instruments (56). As endoscopic technology continues to evolve, endoscopic removal of ascarids has emerged as the preferred treatment method, owing to its safety, speed, minimal patient discomfort, and exceptional outcomes. This approach serves as a valuable educational tool for the diagnosis and management of biliary ascariasis.

### 3.6 Treatment

The preferred medication for deworming is benzimidazole anti-parasite drugs, specifically albendazole (57). It can be administered in a single dose or divided into two doses, with the option to repeat the treatment 10 days after the initial deworming. The medication works by blocking the ascarids’ ability to absorb glucose, resulting in glycogen depletion and reduced adenosine triphosphate production (58). This causes the ascarids to become paralyzed and eventually expelled from the body, typically occurring within 2–4 days following medication. A combination of mebendazole and levamisole, also referred to as compound mebendazole, is an effective broad-spectrum anti-nematode medication. It inhibits neuromuscular transmission, leading to spasmodic contractions and paralysis of ascarids. This facilitates their safe expulsion from the body (29, 58). Additionally, other drugs like piperazine citrate (piperazine) and levamisole also demonstrate satisfactory efficacy in eliminating ascarids (59).

For biliary ascariasis, the treatment regimen includes administering analgesics to alleviate pain, utilizing deworming agents to eliminate the parasites, managing any infections that arise, and correcting any imbalances in water-electrolyte levels and acidosis. In cases of incomplete intestinal obstruction, it is crucial to provide fasting to rest the gastrointestinal tract, decompress the intestines to reduce pressure, and administer analgesics to relieve abdominal pain. Deworming treatment should be administered only once the abdominal pain has subsided. In scenarios where medical interventions prove ineffective or the condition escalates significantly, particularly in cases of complete intestinal obstruction, appendicitis, gastrointestinal perforation, or acute peritonitis, prompt surgical intervention becomes imperative to halt the progression of the illness and prevent its further deterioration (29).

### 3.7 Prevention

To successfully safeguard against ascariasis, we must adopt a comprehensive strategy that encompasses the thorough management of infection sources, rigorous disruption of transmission routes, heightened protection of vulnerable groups, steadfast enforcement of food safety regulations, and consistent promotion of healthy dietary habits (16, 17). Prompt deworming treatment for infected individuals is paramount, while hygiene education must emphasize the importance of thorough handwashing and proper food handling practices. We must discourage defecation outdoors and prioritize proper waste disposal and sewage treatment to minimize fecal pollution. Regular deworming of vulnerable populations, such as children and immunocompromised individuals, is essential, and public education on hygiene measures must be ongoing. Strict enforcement of food safety regulations is vital, and we should consistently promote balanced dietary habits to further reduce the risk of ascariasis transmission. If symptoms like abdominal pain or diarrhea arise, immediate medical attention is imperative.

### 3.8 Debate surrounding gastrointestinal perforation

Although gastrointestinal perforation resulting from AL infection is an exceptionally rare complication, with only 14 documented cases in the previous medical literature, its occurrence is considered highly critical and potentially fatal, necessitating urgent surgical intervention. Nevertheless, the precise mechanisms underlying this complication remain controversial, with two main theories.

One theory, espoused by Efem, is that the perforation caused by the AL is related to ulcers (60). The intestine has a significant expansion capacity, allowing it to accommodate up to 5,000 ascarids without exhibiting any symptoms. Efem maintained that the direct pressure generated by the AL is unlikely to cause intestinal perforation, and there may exist a causal relationship between intestinal perforation caused by the AL and various ulcerative diseases such as typhoid enteritis, tuberculosis, and amebiasis. When the intestinal environment turns hostile due to conditions like starvation, inflammation, or obstruction, the ascarids instinctively migrate to more tolerable regions within the body. They may subsequently traverse into the biliary system, pancreatic duct, or stomach, with the potential of being expelled through the mouth. Moreover, they can block nasogastric tubes, penetrate the peritoneum through ulcerated or surgically sutured tissue, or exit through the anus. While they can exacerbate pre-existing perforations caused by typhoid, amoebiasis, tuberculosis, and other non-specific ulcers, it is highly unlikely that they are the sole cause of perforation in an otherwise healthy intestine. It is noteworthy that, out of the 14 reported cases, only 2 were definitively linked to ulcer disease as a complication.

Subsequently, several reports have presented differing viewpoints, highlighting a common feature among these cases: during the histological examination of the small intestine perforation caused by the AL, no evidence of amoebiasis, typhoid fever, or nonspecific ulceration was found (8–11). This particular type of perforation may stem from the overcrowding of ascarids within the intestinal tract, causing compression that triggers ischemia and necrosis, ultimately culminating in rupture during the ascarids’ movements (13–15).

## 4 Conclusion

Despite the widespread prevalence of AL infection globally, gastric perforation caused by this parasitic infection is a rare but severe complication. Timely diagnosis, surgical intervention, and postoperative management are crucial for successful treatment. Furthermore, this case underscores the importance of maintaining good personal and environmental hygiene habits to prevent the transmission of such parasitic infections.

## Data Availability

The raw data supporting the conclusions of this article will be made available by the authors, without undue reservation.
